# What do geriatric rehabilitation patients and experts consider relevant? Requirements for a digitalised e-coach for sustainable improvement of nutrition and physical activity in older adults – a qualitative focus group study

**DOI:** 10.1186/s12877-021-02692-y

**Published:** 2021-12-18

**Authors:** Lisa Happe, Andreas Hein, Rebecca Diekmann

**Affiliations:** Department of Health Services Research, Assistance Systems and Medical Device Technology, Carl von Ossietzky University Oldenburg, Ammerländer Heerstraße 140, 26129 Oldenburg, Germany

**Keywords:** Technology, E-coach, Geriatric rehabilitation, Older adults, Nutrition, Physical activity, Behaviour change, Focus group

## Abstract

**Background:**

During geriatric rehabilitation, attempts are made to increase the patients’ health and functional capacity. In order to maintain these improvements in the medium- and long-term, behavioural changes regarding mobility and nutrition are also targeted, but these are often not sustainable. International studies show positive effects on the sustainability of the improvement of physical activity behaviour in healthy seniors through the use of electronic devices and software applications. Comparable approaches that include nutrition topics or combine them and were additionally developed for geriatric rehabilitation patients (≥70 years) to date are not known. The aim of this study was to identify what geriatric rehabilitation patients require from an electronic coaching system (e-coach) to support them in improving their nutritional and physical activity behaviour, and what content and features physiotherapists and dieticians consider relevant.

**Method:**

Focus group interviews (09–11/2019) were conducted in a geriatric rehabilitation centre in Germany with patients aged 70 years and older, relatives and experts (physiotherapists and nutritionists). The focus groups were recorded, transcribed verbatim and analysed using content analysis.

**Results:**

Three focus groups with patients and relatives (*n* = 17, 65% female, 16 (94%) in age category 70–99 years) and one focus group with experts (2 dieticians and 1 physiotherapist) were conducted. Relevant contents and feedback elements for nutrition and physical activity in old age were identified. The patients’ comments show that an e-coach must offer obvious benefits for the older persons and promote motivation in order to be used. The willingness to change nutrition and physical activity behaviour and the previous experiences in these areas are very heterogeneous, therefore content should be adaptable to different requirements.

**Conclusion:**

Experts and patients identified quite similar contents, barriers and facilitators for a nutrition and physical activity e-coach. The e-coach needs to be able to address different points of behaviour change, enable adaptations to the individual patient and convince the older person that using it will help them to improve their nutrition and physical activity. It is also important that the e-coach is easy to use and can be easily integrated into the patient’s everyday life after rehabilitation.

**Supplementary Information:**

The online version contains supplementary material available at 10.1186/s12877-021-02692-y.

## Background

Older adults in geriatric rehabilitation facilities are often affected by sarcopenia, malnutrition or are at risk of malnutrition. There is evidence of associations between reduced nutritional status and low physical activity [1]. Sarcopenia and malnutrition are associated with serious negative consequences for those affected. These include a frequent occurrence of the frailty syndrome, an increased risk of falling, physical impairments, reduced quality of life and an increased risk of hospitalisation and mortality [2–4]. Sarcopenia and frailty, on the other hand, can be well treated by specific training and nutritional interventions [3, 5, 6]. For improvement of the nutritional and functional status the patients need to be treated comprehensively, taking into account the patient’s health status, functional capacity and the social context. Geriatric rehabilitation facilities in Europe should have a multidisciplinary team working together with the patients to ensure comprehensive treatment [7, 8].

In order to maintain the patients’ long-term success of rehabilitation interventions, continuing to follow new patterns of behaviour and recommendations after discharge is important. Exercise programmes often fail to ensure the adherence of geriatric patients over a long period of time [9]. Negative influences on adherence are likely to be factors such as sudden changes in health status, lack of interest or motivation, low self-efficacy, weakness or low expectations of improvement. Patients who understood their health problem and the risks involved, and patients who selected the exercises in close consultation with a physiotherapist, showed a higher level of adherence [9, 10]. No data on long-term adherence to dietary recommendations is known for older adults in the nutritional context. However, a study showed that continuation of nutritional counselling in combination with care provided by the general practitioner after a stay in hospital resulted in geriatric patients having a better nutritional and functional status than those who received only general practitioner follow-up care [11].

Alternatively, patients could be supported in the long term by using technical assistance systems (devices like mobile phones or tablets with sensors and software applications (health apps)). Existing health apps to promote activity address community-dwelling older adults who have already developed an awareness of the problem and focus strongly on planning and teaching training programmes rather than offering elements of information and education [12, 13]. In addition, the target groups of previous health apps tend to be older people who are already active in sports groups, who live independently and are not affected by acute health problems [12–14]. An additional integration of nutritional issues does not take place in any of the mentioned apps. The health apps are therefore not directly transferable to the goal of improving changes in the nutrition and exercise behaviour of geriatric rehabilitation patients. Since older people require technical systems that take age related limitations (e.g. visual impairments or difficulties in fine motor skills) into account and also have less experience with and affinity for technology, the use of systems for younger target groups is also not an option [15]. Therefore, an age-adapted device and health app (e-coach) will be developed using the user-centered design approach. In this participatory approach the requirements, views and opinions of the target group are taken into account during the whole development process [16]. Patients should receive the e-coach during rehabilitation and become familiar with its use. After rehabilitation, patients should continue to use the e-coach and thus be supported in maintaining the new behaviours.

This study is the first step in the user-centered design approach for the e-coach. We performed focus groups with geriatric rehabilitation patients and experts for the context analysis of the target group. Within this study attitudes, experiences and lifestyles of the target group will be explored and information on specific contents and requirements for the system will be defined.

## Methods

Three focus groups with geriatric rehabilitation patients and their relatives and one with experts (physiotherapists and dieticians) were conducted from September 2019 to November 2019. The study was approved by the Ethical Review Board of the Carl von Ossietzky University Oldenburg (registration number: 2018–131). We conducted the study in accordance with the Declaration of Helsinki [17] as amended and the underlying data protection regulation. Reporting followed the COREQ checklist for interviews and focus groups [18].

An interview guide that includes the following topics was developed for the focus groups:

1. Relevant topics from the areas of nutrition and physical activity for geriatric rehabilitation patients and possibilities of presentation.
2. Content and presentation options for feedback on physical activity and nutrition on a tablet screen.
3. Facilitators and Barriers for the use of the e-coach.

The guide was pre-tested by a pilot focus group with community-dwelling older adults and minor adjustments were made.

### Participants

All participants were recruited from a geriatric rehabilitation centre in the north-western part of Germany. The experts were working at the department of physio- or nutritional therapy of the rehabilitation centre and were named by the heads of the departments for participation in the focus group.

The inclusion criteria for the older people were the following: (1) minimum age of 70 years, (2) geriatric stay in the study rehabilitation centre. Exclusion criteria were (1) significant cognitive impairment, (2) severe hearing impairment, (3) severe visual impairment, (4) severe mobility impairment (bed bound), (5) German language barrier. Geriatric rehabilitation patients were recruited by flyers placed at the information board at the patient’s wards. Interested patients received detailed information on the content and procedure of the study in a personal meeting with LH. Patients who were eligible for the study and gave written informed consent were also informed of the possibility of bringing a relative to the focus group discussions. When at least four older people had declared their willingness to participate, a date for a focus group was set. This date took place during the stay in the in-patient rehabilitation centre and was communicated to the participants personally in advance, but also appeared on their event and therapy schedule that day.

### Data collection

#### Procedure

Prior to the focus group meeting, demographic data (gender, age category) was surveyed (Table 1). In addition, the technical commitment was assessed with the questionnaire published by Neyer et al. in 2012. In this questionnaire, the technology commitment is measured using a 5-point Likert scale with 12 items. The items are statements about personal contact, interest and use of technologies in general. In the evaluation, depending on the item’s polarity, the possible answers are assigned a value between 1 and 5 points. The questionnaire can be divided into the areas technology acceptance, technology competence and technology control [19].

**Table 1:** Overview of participants characteristics in the patient focus groups

Group 1			Group 2			Group 3		
Gender	Age Group	TC^**a**^ Points, mean ± SD	Gender	Age Group	TC^**a**^ Points, mean ± SD	Gender	Age Group	TC^**a**^ Points, mean ± SD
Female	80–84	2.6 ± 1.0	Male	85–89	2.9 ± 1.2	Male	95–99	3.8 ± 1.1
Female	75–79	3.2 ± 1.9	Female	75–79	3.1 ± 1.7	Female	80–84	3.7 ± 1.4
Male	80–84	2.3 ± 1.2	Female	80–84	2.9 ± 1.2	Female^c^	< 70	2.8 ± 1.1
Male	70–74	2.3 ± 1.6	Female	70–74	3.5 ± 1.0	Male	80–84	2.7 ± 0.9
			Male	85–89	2.5 ± 0.9	Female	80–84	3.4 ± 1.0
			Male^b^	80–84	1.9 ± 0.6	Female	80–84	2.9 ± 0.9
						Female	80–84	3.2 ± 0.8

^a^*TC* Technology commitment; ^b^ Relative (Husband); ^c^ Relative (Sister)

The focus groups were held in a room in the geriatric rehabilitation centre where normally the group therapy sessions and nutrition seminars took place. Initially participants were greeted, offered something to drink, and asked to sit down at a table. All focus groups were led by the same moderator (LH) to ensure uniformity between focus groups [20]. The moderator is a physiotherapist herself and PhD student in a working group on nutrition and physical function in old age. In preparation for the moderation of focus groups, she has observed focus group discussions with an experienced researcher who has already conducted focus group studies with older people on several occasions. She also conducted the test focus group and received supervision there as well. In addition, an assistant moderator was responsible for note taking on different thematic posters. All focus group interviews started with an introduction that explained the background of the project, the methodology and the reasons for the involvement of LH. It was emphasised that the main purpose of the meeting was the discussion and dialogue between the participants. Participants were encouraged to openly present their views and discuss with one another. The whole group was always addressed and an attempt was made to give all participants the opportunity to contribute to the discussion to the same extent [20].

#### Content focus

After the moderator’s introduction, the participants were asked to introduce themselves to the group. Next, the participants were asked to discuss the following introductory interview questions: (i) On which aspects of nutrition and physical activity would you find it helpful to obtain more information and how can it be presented? (different forms of presenting information by text, graphic and video were shown as key stimulus), (ii) How should the screen be structured to provide you with feedback on your physical activity/nutritional behaviour? (two variants from feedback screens for each topic were shown as key stimulus) (Additional file 1), (iii) What could motivate you to use such an e-coach, what could be a hindrance (i.e. operating the e-coach, support, design)? All interviews were audio taped and lasted between 53 and 62 min.

### Analysis

The audio recordings were transcribed by one person in the form of an extended semantic transcription. Pseudonyms were assigned to each patient and all identifiable features were deleted. The transcriptions were then checked by an uninvolved person on the basis of the audio recordings and discrepancies were discussed. The data from the focus group interviews was analysed in the form of a structured qualitative content analysis according to Kuckartz [21]. First, a priori main categories derived from the interview guideline were developed. During the coding process, subcategories were inductively formed. The computer-aided coding of the text segments into the categories was performed using the program MAXQDA 2018. The entire coding process was done by two persons independently. The categories were critically discussed by the two researchers until consent was reached.

## Results

Out of 43 patients contacted, 16 decided to participate; one did not attend the focus group finally. A total of 15 patients and two relatives (10 women, 7 men) participated in three focus groups with patients. The number of participants varied between 4 and 7 participants per focus group.

Technology commitment was at a median of 2.9 ± 1.2 points. For the different subscales, technology acceptance showed the lowest level with a mean value of 2.2 ± 1.1 points.

Four therapists (two nutritionists and two physiotherapists) agreed to participate in the expert focus group. However, one physiotherapist was unable to attend due to illness. The three participating therapists were all female, one was in the age category 25–30 years and two were in the age category 35–40 years.

In the evaluation of the focus group discussions, five main categories with 19 subcategories were identified (Table 2). The analyses of the identified categories are presented in accordance with the focus group interview guide.

**Table 2:** Categories and subcategories from the focus groups

Category	Subcategory
Contents of the information provision	Physical activity topics
	Nutritional topics
Forms of information distribution	Design of educational elements
	Layout of the screen for nutritional feedback
	Layout of the screen for physical activity feedback
Barriers to the use of the e-coach	Personal interest
	Integration into daily life
	Dealing with technology
	Barriers due to the use of individual strategies
	Design aspects
	Design of exercises
Motivating factors	Motivation through personal interest
	Motivation through contents of the e-coach
	Motivation through social aspects
Helpful features and settings	Own existing technical know-how
	Design and construction of the e-coach
	Issues related to the exercises
	Individual support while using the system
	Support by the program

### Physical activity topics for the e-coach

Patients considered an e-coach as an opportunity to be instructed in exercises. The requirements for these exercises were adaptability to different performance levels and illness-related limitations, flexibility in scheduling and execution, and integration into everyday life. Exercises that could be done in the community or together with the neighbour would also be advantageous, as the participants would be more motivated. The participants themselves saw a need for information on topics such as strategies for maintaining exercise programmes in the long term. Factors influencing falls in old age were discussed, but there was a clear need for further information on this topic:

*There are so many people falling, I ask myself is it because they don't wear proper shoes or what is the reason for that?*

(Participant 1, Focus group 3)

Also, a need for more information on other themes for example the relevance of training certain muscle groups was demonstrated. The use of the walker was mentioned by several participants. For example, one participant found it very helpful to use the walker and thus regained a bit of quality of life. Other participants said that they had the feeling of being dependent on the walker and thus lose their balance rather than gain more security. The correct handling of the walker and reasons for which a rollator might be used should therefore be considered as a part of the e-coach.

The experts mentioned information on exercises with consideration of existing limitations and adaptation to the individual patient as possible elements. In addition, they discussed that warnings should also be part of the e-coach, for example what to do if problems occur during exercises. They also mentioned different forms of additional feedback or reminder functions as features of the e-coach. A reminder could inform the participant on regular exercise execution or the need to drink something, especially if the patient is also physically active. In addition, the e-coach could provide information on local services. It was reported that patients often asked about possibilities where they could continue physical activities in the patients’ residential areas after rehabilitation, e.g. senior gymnastics groups. The therapists would find a feature that offers this information very helpful, because they didn’t have a platform or something where they could find information on physical activity classes near the patient’s residential area, especially if the patient lived in a different city.

### Nutritional topics for the e-coach

To varying degrees, patients already had knowledge about nutrition in old age. Topics such as varied diet, sufficient drinking amounts and the importance of macronutrients were already known by many. However, the discussion of these issues identified some gaps in knowledge, for example about how energy requirements change with physical activity or about weight loss in old age. In addition, each focus group addressed the topic of nutrition myths, and it became clear that the participants were very concerned and insecure about these myths:

*Yes, you’ re supposed to eat a lot of protein-rich food, um, and um, one says: 'Not so much meat', the others say: 'Yes, you can eat meat', that's always / (.) Everyone says something different, right?*

Participant 1, Focus group 1

Another point of discussion was the preparation of food and the respective changes in older age. Some participants described that they had tried to change their diet, but old habits and the environment (partner) hamper. Suggestions for recipes might help them, but they also said that it is difficult if the relatives do not accept the new recipes. Furthermore, the participants had experienced that it can be difficult to supply themselves with food in old age. Nutritional supply can be impaired by mobility restrictions or loneliness, when he or she was used to cooking for several persons or someone else always prepared the food so far.

The experts identified information about protein consumption, sufficient liquid supply in old age, as well as examples of snacks as important issues for the e-coach. Another idea by the nutritionists was to provide simple recipes in form of a written recipe or a cooking video to give examples for the preparation of protein-rich meals. The nutritionists reported that older adults had problems in obtaining food because of mobility restrictions, visual impairment or infrastructural difficulties, e.g. when in rural areas the nearest supermarket is in another village. Also, older adults often asked for information on possibilities where they can get a ready cooked meal when they had problems with the food preparation or simply don’t want to eat alone. A strategy to address these problems could be a digital map of the place of residence in the e-coach with information on support like shopping facilities where help for older people is offered, open lunches or meal delivery services specially for older people.

### Forms of information distribution

Information could be provided in form of texts. This is already known for educational elements and could be used, for example, to communicate nutritional myths. A large font is important here. The participants were already familiar with the combination of text and images from seminars and training courses. Educational videos were also already known by some participants and appear to be an acceptable form of information provision:

*I would say that if it is presented pictorially and explained very well, I would say then one could consider whether to follow it.*

Participant 5, Focus group 3

Participants found it helpful to have an exercise “demonstrated by a person” and to be able to call it up at any time visualise the exercise again. They stated that the preferred way of providing information depends on the individual preferences, but that a clear presentation and good comprehensibility should always be considered regardless of the form of presentation.

Some patients showed difficulties in interpreting and understanding the nutrition feedback screens (Additional file 1). They mentioned not to be familiar with the term protein or energy as a reason. Other participants said that they never needed electronic feedback on nutrition or physical activity in general and therefore found it difficult to understand these screens. The discussion about feedback options clarified the following: patients want to be able to see directly what the goal of energy and protein intake is and how much they had already achieved. In an exercise diary, photographs of a person performing the exercise are preferred over illustrations (Additional file 1). Exercises that had already been completed should be marked, for example with a tick. Feedback on the number of steps should include the total number of steps and information on how much is still missing to reach the goal.

Experts preferred to provide most information as video. A clear background should also be used for exercise photos in the exercise library and movement arrows should be integrated in these photos. Exercises should be demonstrated by a real person in a video, which should not contain any disturbing variables (simple clothing, no distracting background) and should be acoustically recorded. During the exercises, it should be possible to give feedback on intensity and difficulty. Graphics should be very clear, not cluttered. In addition, patients should receive visual feedback on both the amount of fluid and the amount of nutrients they consumed. The information on local services (e.g. senior gymnastics groups, open lunches) should be provided by an interactive map that refers to the patient’s residential area.

### Barriers to use the e-coach

Patients didn’t want to be patronised or receive too strict rules from the e-coach. The use of the programme must not limit them in their everyday life or be time-consuming and cause stress. They also must be convinced of the advantages and benefits of the e-coach. Patients saw their lack of technical experience due to the lack of use of technology in their previous working life as a possible barrier. Some patients reported having bad experiences with learning new technologies. They have had some experience that they cannot follow the explanations and that it seems too complicated to them. The experience has also been reported that younger relatives often become impatient when explaining new technologies to older adults.. The system should not require many different skills and should not put pressure on them. Patients emphasised that they would only use the e-coach in the long term if they had their own motivation and interest:

*In old age, too, it always depends on what you have to do. So, if I want to do something, that is something different. Right? But I have also reached an age where I can say: `Why should you then / why should you still get yourself into it´, right?*

Participant 5, Focus group 3

Patients mentioned as design barriers a small font, too fast running videos and small buttons. When people are shown in videos or graphics, it was also important that older people can identify with their appearance. Exercise videos should show safe execution of the exercise and also provide advice on safe execution. For example, if the video recommends supporting during the exercise, the shown object used for support should be very stable..

The experts thought that the biggest barrier was the low technical skills of the target group. Considering graphical design, the experts mentioned, that long texts could be discouraging and overstrain visually impaired people. Visual design elements such as long texts, distracting elements or unclear, confusing presentations are barriers that could hinder use of the e-coach. The experts would be hampered to use the e-coach with the patients when it is very time consuming and if the use stops the patients from being physical active.

### Motivating factors

In order to motivate people to do exercise regularly, it should be related to everyday life, several body regions should be trained and exercise parameters (e.g. number of repetitions) should be variable. The use of visual elements could support the motivation to deal with information content. The possibility of using the programme in a group or together with others could also increase the motivation to use the e-coach. Participants reported that their children and grandchildren motivated them most to use technology. An encouragement for long-term engagement could help to motivate, such as gamification elements e.g. points or stars collected for completed tasks or feedback on the patient’s input and use:

*[…] but also because I can check it on the tablet in the evening and then I'm proud of myself: `I have achieved that´.*

Participant 4, Focus group 2

The experts assumed that different feedback content for the patient and also therapists could increase the motivation to use the system (for example, feedback on exercise intensity, drinking protocol, pain intensity) is desired. In addition, patients could be offered a reward system in the e-coach for achieved physical activity goals. A feature of which the experts also thought it could motivate the participants was the possibility of connecting participants with another and by this motivate them to stick to their goals, training programs and food recommendations together.

### Helpful features and settings

Some participants already had experience in using new technologies (e.g. fitness trackers or e-mail). However, they said very clearly that the advantage over “conventional” methods must be obvious if they should use the e-coach. For both technical content and elements, it could be helpful if it is based on known technical actions. The effort required to operate the system should be kept to a minimum and should be adapted to the individual user’s skills and wishes. An introduction to the use of the e-coach should take place in a one-to-one setting.

Exercises and goals should be selected in cooperation between patients and physiotherapists and individual adaptation should be possible:

*Everybody does a programme on their own, right? As he likes it best. Whether he wants to do ten exercises a day or only three exercises a day.*

Participant 1, Focus group 2

A reminder function, if no input has been made for a longer period of time, was considered as possible feature. The e-coach should be adapted to the needs of seniors by a large font size, large buttons and images and an appropriate response time. Presentation and evaluation elements should be easy to understand at a glance and have a clear labelling.

A helpful function of an e-coach mentioned by all experts was the possibility to adapt it to the individual patient. Therefor the input of biometric data, secondary illnesses, walking aids, preferences (e.g. food preferences) and previous experiences (e.g. already regular performed physical activities like strength training) should be an option. Moreover, the experts referred to design aspects like big text sizes and higher volume levels as helpful contents to make the e-coach usable for older people. For the physiotherapists it would be helpful if the e-coach offers a library of different exercises from which the therapist could choose exercises for the patients training schedule in the e-coach.

## Discussion

This study was the first step in the user-centred design process for a nutrition and physical activity e-coach for geriatric rehabilitation patients to support a sustainable behaviour change in these areas. The results of this study provide insights into the future context of use of the e-coach. Relevant nutritional and physical activity topics were identified and compared with the experts’ impressions. In order to facilitate the applicability of the programme and its long-term use, various requirements were identified and discussed by the participants.

In the discussion of relevant topics for the e-coach, both patients and therapists mentioned very similar points. In the area of nutrition, there was a lot of debate about the fact that older people need information about protein-rich foods and a balanced diet. The individual perception of the need for further information varied widely. Some participants said that they needed more information on certain issues, while others said that they already knew a lot about nutrition. This heterogeneity of nutrition knowledge was also reported in a large multicentre study involving people over 65 years of age, in five European countries. In the study, associations were also found between higher knowledge of nutritional issues and higher levels of physical activity and younger age (< 75 years) [22]. In all focus groups it was noticeable that there was a lot of uncertainty about some topics, such as nutrition myths e.g. the right, respectively wrong consumption of eggs and milk products, or weight loss in old age. These identified knowledge gaps should be taken into account when developing modules for the e-coach. Different factors that negatively influence the consumption of protein-rich animal-based foods in older adults have already been investigated in other studies [23–25]. These studies also identified misconceptions and lack of knowledge as reasons for lower consumption of animal-based foods. Misconception and lack of knowledge can be reduced, but it is important that the information is clear, short, simple and specific [23]. Egg consumption, in particular, seems to be a contentious issue among older people, as the discussion content of our focus groups also shows. For example, in a survey of older people in the United Kingdom, eggs were among the least commonly eaten animal products. Ways to influence this aspect of nutrition in specific, in addition to the education already described to reduce misconceptions, include practical interventions such as teaching how to cook dishes to improve tastiness and increase variety, as well as further information about storage and shelf life [24, 25].

Other reasons that make it difficult for older adults to change their dietary behaviour are described in the study by Best et al. (2013). One factor we also found in our focus group discussions was that older, widowed men in particular often have problems with cooking. Participants in our focus groups described that it usually was the women who were responsible for preparing the food and that men had only very rudimentary cooking skills and did not feel confident to cook. Thus, it is important for these people either to be shown alternative ways of obtaining meals, e.g. community meals and special delivery services for older people, or to be taught simple, precisely explained recipes. Another influencing factor that we also found in our discussions was that due to poor mobility and physical disabilities, both the shopping for groceries and the preparation of meals is more difficult. In addition to the already described alternative ways of obtaining food and providing simple recipes, advice on food storage could also be helpful. A factor that was less discussed in our focus groups and which Best et al. (2013) describe as difficult to influence is that some older people eat less or no longer eat certain foods due to sensory changes such as a reduced olfactory capacity and a reduced sense of taste [23]. To address this problem, especially with regard to protein-rich foods, the e-coach could, for example, point out alternatives to certain foods with which it is possible to compensate for certain food groups.

Also, in the area of physical activity some issues were identified where uncertainties or gaps in knowledge seem to exist. For example, the risk of falling in old age and the influence of environmental factors such as falling hazards or the use of walking aids were among these issues. Information on the relevance of sufficient fluid intake and how to increase it was identified as an important element in both contexts: nutrition and physical activity. In order to eliminate the existing misinformation and uncertainties, the target group should be provided with current evidence-based knowledge. The evidence should be presented in a patient-oriented form with the necessary background information and provided via the e-coach.

Regarding physical activity, many patients have reported that it is difficult to maintain new training routines. Therefore, it seems very important that an e-coaching programme addresses these issues and helps them to maintain these routines in the long term. Furthermore, many patients said that they would find it helpful if exercises were taught with the e-coach. The patients were able to describe very precisely what requirements they had for an exercise programme, e.g. adaptability to different performance levels or physical limitations. These requirements of older people for an exercise program taught through a technical system are very similar to results from other qualitative studies. The participants from these studies were older adults who were already engaged in regular physical activities and had no acute health issues. In one study examples of web-based physical activity programmes were shown to the participants and in the other study participants were interviewed after participation in a web-based training programme and also people who refused to take part in this training programme [26, 27].

Overall, the participants’ knowledge levels, personal needs and interests in the areas of nutrition and physical activity were very heterogeneous. If people are supposed to change their behaviour, it is important that they first understand what the problem is and why it might have negative effects. If this awareness is already present, appropriate strategies can support the planning, implementation and maintenance of new behaviours [28, 29]. The e-coach should enable adaptations to the individual patient and accompany the behaviour change process by using appropriate strategies with an underlying psychological concept. In the patient focus groups, it was noticeable that there appears to be a general difference in the acceptance of interventions to improve diet and physical activity among this age group. While physical exercise and the need for physical activity were considered useful and necessary by most participants, there seems to be more scepticism or aversion to patronisation when it comes to the willingness to change the diet. When communicating recommendations and feedback on the dietary situation, special attention should be paid not to convey negative emotions or constraints.

The median technology commitment among the participants was 2.9 ± 1.2 points. This value is comparable to the results of Rasche et al. (2018) on the technology commitment of older adults who do not yet use apps (2.9 ± 0.6 points). Within the scope of the study, older people were asked about the use of apps in general and health apps. The participants who already used apps scored significantly higher (3.4 ± 0.6 points). The survey by Rasche et al. (2018) also examined barriers to the use of health apps. Barriers included lack of trust in health apps, lack of self-confidence, data security concerns, concerns about misdiagnosis and poor usability of health apps [30]. These findings are also reflected in the results of our focus groups, with participants clearly stating that they need to be convinced of the benefits of using an app and the app itself before using it. A study from Sweden on attitudes and beliefs about e-health services and expectations on e-health tools also showed that the motivation of older people to use e-health structures is only existent if there is experience of difficulties in current care or awareness of the advantages of an e-health solution over standard care. Participants from this study were older adults (65–80 years old) and recruited from primary health care facilities in different socioeconomic areas in Sweden, had at least one chronic disease and were treated with at least three drugs continuously [31].

If the older people are going to deal with new technology, they need detailed instruction in how to use the e-coach and the tablet. The instruction should be given in a one-to-one setting and should not be overwhelming. If the participants continue to use the device after their rehabilitation stay, it must be possible to integrate it into their everyday life without being too time-consuming, so that individual life is not restricted by the recommendations and no stress is produced by the use. This need for good integration of new technologies into everyday life is also reflected in the results of other studies with community dwelling older adults. In these studies, web-based physical activity programmes were presented or the participants were interviewed following the use of a health app or a web-based exercise program. The participants already had some experience with technology, the internet and also had technical devices such as computers or tablets [26, 27, 32].

Analysis of design concepts, the results of other focus group studies with older people and the results of our study address the particular relevance of developing a system taking age-related limitations and low complexity into account. For example, a sufficient font size should be used, the handling should be simple, the screens should not be cluttered and in case of videos the volume should be high enough [26, 32, 33]. It would therefore make sense to use a larger screen, such as on a tablet. Thus, the relevant design aspects such as sufficient font size, size of the buttons and sufficient spacing between individual screen elements can be ensured without cluttering the screens.

The results of this study should be interpreted with the following limitations in mind. The participants in the different focus groups were not stratified by age or gender. In Germany a geriatric rehabilitation programme lasts about 3 weeks. Participants for this study had to be recruited during this period and an appointment had to be planned in addition to the treatments and seminars taking place in the institution. As there were at least four participants per focus group needed at every time to allow real discussion and exchange of views, and recruitment usually resulted in a maximum of three participants per week, no further separation according to age or gender was practical. However, it cannot be ruled out that homogeneous focus groups of older adults would have generated different responses and opinions, even though our focus groups did not address sensitive or gender-specific issues.

Moreover, it is likely that participants in the focus groups were already interested in the topics of nutrition and physical activity in old age or had a stronger interest in technology than older people who have less experience or interest in these topics. A selection bias can therefore not be ruled out.

In the focus group discussions, the moderator always tried to involve all participants equally in the discussions, and quieter participants were actively encouraged to participate through personal address. However, it is possible that individual opinions or ideas were not shared during the discussion and also that very active participants were able to focus more on their opinions than less active persons.

## Conclusion

By focus group discussions relevant topics, barriers and helpful factors for a nutrition and physical activity e-coach for geriatric rehabilitation patients have been identified. Since many patients in this age group have little experience with technology and mostly used other sources of information, it is important that the development of a nutrition and mobility e-coach considers particularly an easy handling and provides clearly the advantages for the individual user. The content needs to be adaptable to the awareness of a need of behaviour change and physical abilities of the users. Moreover, the e-coach needs to be integrated into their daily life without stressing or restricting its users. Further development of an appropriate e-coach system should be done in close cooperation with the target group in order to properly implement these various requirements and to create an e-coach with a high degree of user-friendliness.

## Supplementary Information

**Additional file 1.**

## Data Availability

The datasets analysed during the current study available from the corresponding author on reasonable request.
