# Supplementary material for: What do geriatric rehabilitation patients and experts consider relevant? Requirements for a digitalised e-coach for sustainable improvement of nutrition and physical activity in older adults – a qualitative focus group study
Source: BMC Geriatr. 2021 Dec 18;21:712. doi: 10.1186/s12877-021-02692-y (PMC8684219; doi:10.1186/s12877-021-02692-y)
Supplement: Supplementary file 1 — Additional file 1. [file 12877_2021_2692_MOESM1_ESM.pdf]

Feedback Screens Examples Nutrition Diary

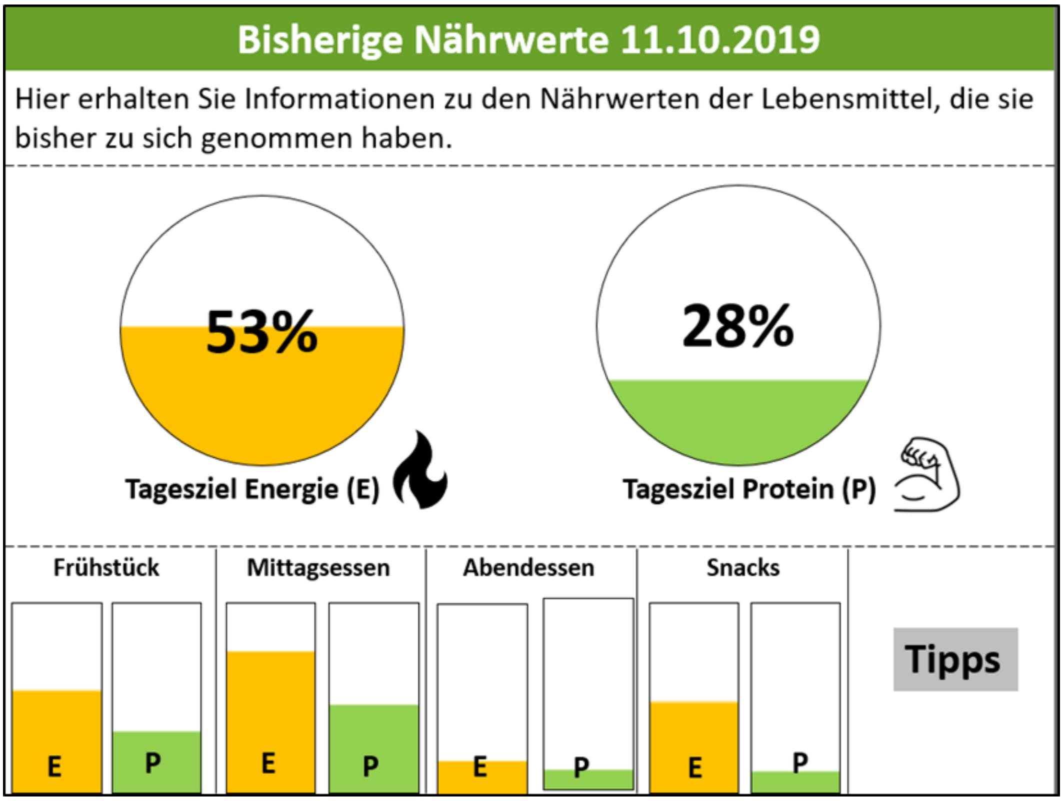

Feedback Screen Nutrition Diary 1

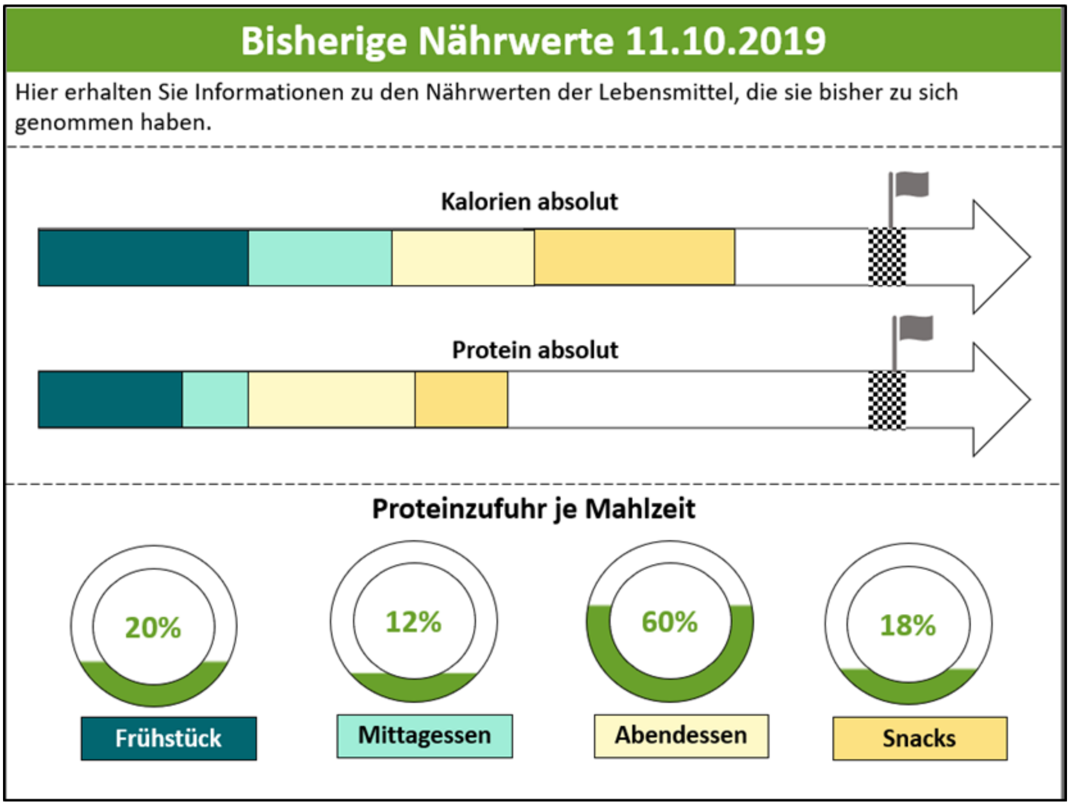

Feedback Screen Nutrition Diary 2

Feedback Screens Examples Physical Activity

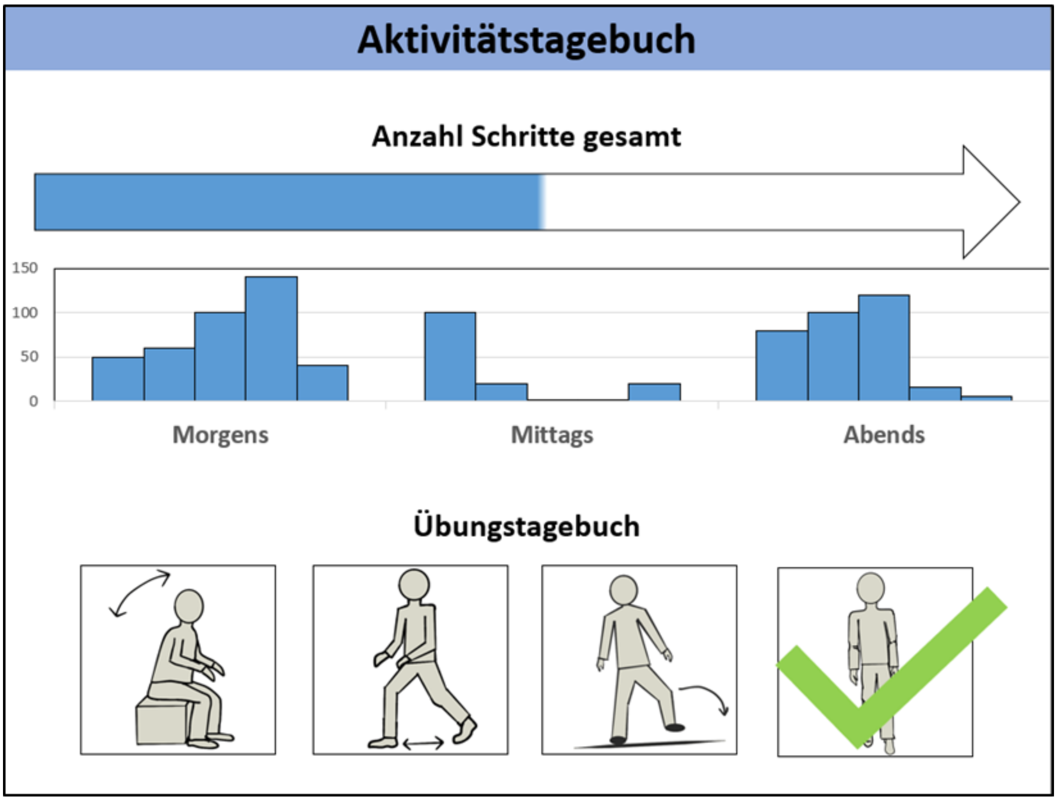

Feedback Screen Physical Activity 1

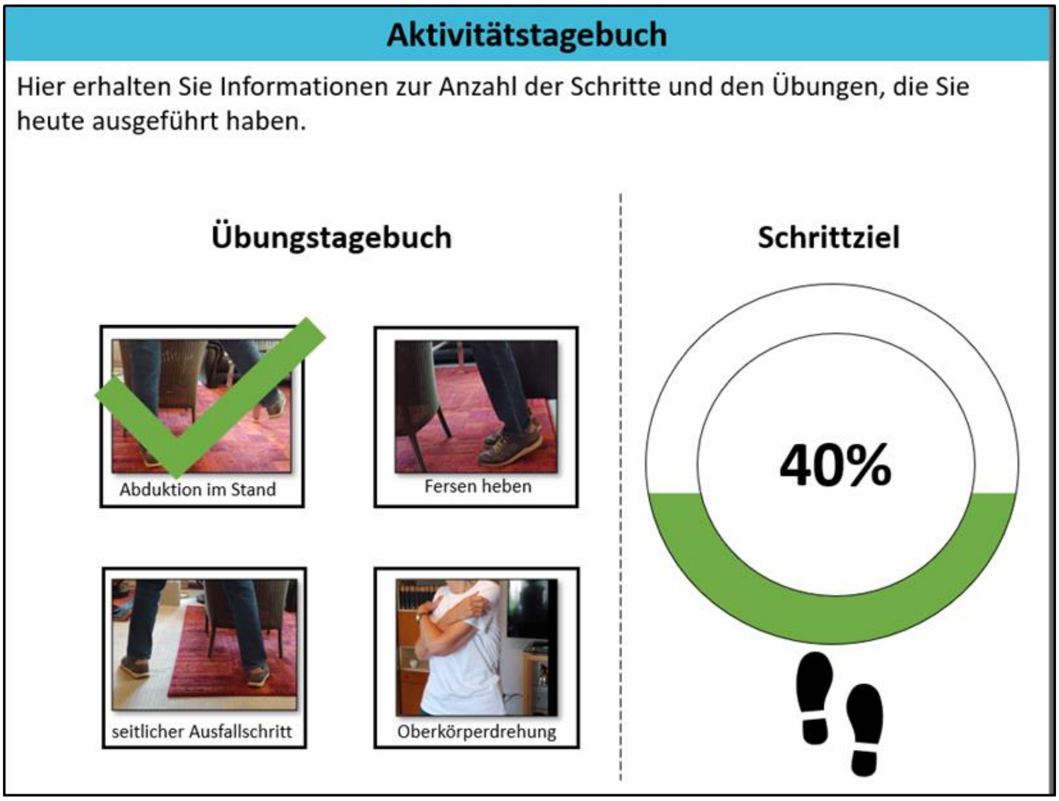

Feedback Screen Physical Activity 2
